# Long- versus short-interval follow-up of cytologically benign thyroid nodules: a prospective cohort study

**DOI:** 10.1186/s12916-016-0554-1

**Published:** 2016-01-27

**Authors:** Marco Medici, Xiaoyun Liu, Norra Kwong, Trevor E. Angell, Ellen Marqusee, Matthew I. Kim, Erik K. Alexander

**Affiliations:** The Thyroid Section, Division of Endocrinology, Diabetes and Hypertension, Brigham and Women’s Hospital and Harvard Medical School, 75 Francis Street, PBB-B4. Room 417, Boston, MA 02115 USA

**Keywords:** Benign thyroid nodule, Follow-up, Thyroid cancer, Thyroidectomy

## Abstract

**Background:**

Thyroid nodules are common, and most are benign. Given the risk of false-negative cytology (i.e. malignancy), follow-up is recommended after 1–2 years, though this recommendation is based solely on expert opinion. Sonographic appearance may assist with planning, but is limited by large inter-observer variability. We therefore compared the safety and efficacy of long- versus short-interval follow-up after a benign initial aspiration, regardless of sonographic appearance.

**Methods:**

This study evaluated all patients referred to the Brigham and Women’s Hospital Thyroid Nodule Clinic, between 1999 and 2010, with a cytologically benign nodule >1 cm and who had returned for follow-up sonographic evaluation. Despite standard clinical recommendations, variation in patient compliance resulted in variable follow-up intervals from time of initial aspiration to the first repeat evaluation. Main outcome measures included nodule growth, repeat fine needle aspiration (FNA), thyroidectomy, malignancy, and disease-specific mortality.

**Results:**

We evaluated 1,254 patients with 1,819 cytologically benign nodules, with a median time to first follow-up of 1.4 years (range, 0.5–14.1 years). The longer the follow-up interval, the more nodules grew and the more repeat FNAs were performed (*P* <0.001). The most clinical meaningful endpoints of malignancy or mortality, however, did not differ between the various follow-up intervals. The risk of a thyroidectomy (usually because of compressive symptoms) increased when time to first follow-up exceeded >3 years (4.9 % vs. 1.2 %, *P* = 0.0001), though no difference in malignancy risk was identified (0.2–0.8 %, *P* = 0.77). No (0 %) thyroid cancer-specific deaths were identified in either cohort.

**Conclusions:**

While expert opinion currently recommends repeat evaluation of a cytologically benign nodule at 1–2 years, these are the first data to demonstrate that this interval can be safely extended to 3 years without increased mortality or patient harm. Nodule growth can be expected, though detection of malignancies is unchanged. While replication of these data in large prospective multicenter studies is needed, this extension in follow-up interval would reduce unnecessary visits and medical interventions for millions of affected patients worldwide, leading to healthcare savings.

Please see related commentary article: http://dx.doi.org/10.1186/s12916-016-0559-9 and research article: http://dx.doi.org/10.1186/s12916-015-0419-z.

## Background

Thyroid nodules are increasingly common, affecting 5–20 % of the adult population [1–3]. Once detected, ultrasound (US) is recommended, often in conjunction with fine needle aspiration (FNA). A benign cytological result is obtained in 65–75 % of cases, most often leading to a recommendation for conservative (i.e. non-surgical) management. However, cytological examination is not flawless and many studies confirm a modest, though non-negligible 1–10 % risk of a false-negative cytology (i.e. malignancy) [4]. Because of this, physicians have long recommended continued follow-up of cytologically benign nodules [5, 6].

However, the empirical evidence for this recommendation remains weak, as there are little data on the optimal time interval for such a repeat evaluation, while other investigations have suggested a negligible risk of disease-specific mortality regardless of follow-up strategy [7]. Current guidelines recommend repeat evaluation after 1–2 years; however, this is based solely on expert opinion [5, 6]. Furthermore, it is unclear what specific findings at the time of repeat evaluation should prompt concern or further investigation, which has led to confusion and variability in clinical practice.

The importance of establishing improved guidance should not be underestimated. Thyroid nodules afflict millions of patients worldwide, and most prove benign. Despite this, follow-up and repeat assessment are extended over many years, with some undergoing lifelong follow-up. Nodule growth can be expected over time, though most often it does not signal malignant concern [8, 9]. While US is superior to physical examination of the neck [10, 11], such technology also exacerbates concern regarding small changes in nodule size or parenchyma. In an era of increasing healthcare cost and intervention, some have argued that overtreatment and overdiagnosis of nodular thyroid disease is occurring [2, 12, 13]. It is estimated that over 500,000 thyroid nodule aspirates occur annually in the United States alone (Medicare data) [14]; as 300,000–350,000 will prove benign, millions of repeat assessments are mandated during the years thereafter. Determining the optimal time interval for such evaluations would reduce unnecessary anxieties, while likely leading to fewer US examinations, repeat aspirations, and thyroidectomies. Ultimately, patient morbidity may be decreased while healthcare savings are realized.

At Brigham and Women’s Hospital, a prospective database has been maintained from 1995 onward, tracking all patients evaluated for nodular thyroid disease using US examination and US-guidance for FNA. This affords a unique opportunity to address this important follow-up question. Specifically, while each patient with initially benign cytology was asked to return for repeat thyroid assessment in 1 year, patient compliance with this recommendation varied. Thus, some patients returned for repeat sonographic assessment in less than 1 year, while others were reassessed after much longer periods. Though non-randomized, such variation is free of substantial referral bias. We therefore used this opportunity to determine differences between short- and long-interval follow-up strategies after initial benign cytology.

## Methods

We reviewed the medical records of all patients referred to the thyroid biopsy clinic of the Brigham and Women’s Hospital, Boston, MA, between January 1999 and January 2010. We identified all euthyroid patients with a clinically-relevant thyroid nodule (>1 cm) found to have initial benign cytology. Previous analysis confirmed that this clinic evaluates >95 % of all patients seeking thyroid nodule evaluation in our healthcare system, thus limiting referral bias. The Brigham and Women’s Hospital catchment generally includes the greater Boston area and southwestern Massachusetts, including some patients from Rhode Island and Connecticut. US evaluation was performed by one of four radiologists with expertise in thyroid evaluation, using a 6–15 mHz transducer (GE Logic 9, GE Healthcare, Milwaukee, WI). The length, width and depth of each nodule was documented, in addition to its solid or cystic content. FNA was performed by one of four thyroidologists under US guidance. A 25-gauge needle was typically used to obtain three samples per nodule, and FNA cytology was evaluated by a Brigham and Women’s Hospital cytopathologist. Although much of the study predates the Bethesda System for Reporting Thyroid Cytopathology [15, 16], all Brigham and Women’s Hospital cytopathologists were already using identical criteria later adopted by the Bethesda System for Reporting Thyroid Cytopathology. All thyroid FNAs were classified into one of the following categories: non-diagnostic, negative for malignant cells (benign), atypical cells of undetermined significance (AUS), suggestive of a follicular or Hurthle cell neoplasm, suspicious for malignancy, or positive for malignancy. The vast majority of cytologically benign nodules were followed conservatively, with regular sonographic assessments. Patients were typically asked to return for repeat thyroid assessment in 1 year.

For the purpose of this study, we took advantage of the known variability in patient compliance with the above recommendation, specifically acknowledging that some patients return for repeat assessment in less than 1 year, while others were reassessed after much longer periods. We therefore identified all patients with cytologically benign thyroid nodules who pursued repeat sonographic evaluation at some point following the diagnosis of a cytologically benign thyroid nodule, regardless of time interval. Each patient was analyzed as a single subject with a follow-up interval defined as the time between first benign aspiration and their first repeat sonographic evaluation, regardless if subsequent ultrasounds were performed thereafter. This is because management decisions made at subsequent follow-up visits are likely influenced by previous visits, while our goal was to investigate the clinical outcomes as a function of various time intervals through the first follow-up visit. Patients whose first follow-up was less than 6 months after initial benign aspiration were excluded, as such scenarios were deemed unique and likely influenced by separate factors.

Nodule measurements were documented at baseline and at the follow-up visit. Other follow-up variables were also collected, including if a repeat FNA or if thyroidectomy were performed. We used our Electronic Medical Record system to identify whether each patient was presently living or deceased (and if so, the cause of death) to determine if the detection of a malignancy at a delayed follow-up period was associated with increased disease-specific mortality. This protocol was approved by the Investigational Review Board of the Brigham and Women’s Hospital (IDs 1999P002899 and 2000P000167). Individual written consent was not required given the lack of intervention and reporting of data in an individually non-identifiable way.

### Statistical analyses

The follow-up time between the two visits was rounded to the nearest whole month. We grouped subjects according to follow-up time into one of five clinically relevant quintiles as follows: 0.5–1 year, >1–2 years, >2–3 years, >3–4 years, and >4 years. To determine change in size with time, nodule volume was calculated by using the formula for a rotational ellipsoid (length × width × depth × π/6) [17]. We employed two commonly used criteria for defining sonographic growth over time. The first was a volume increase of 15 %, while the second was a volume increase of 50 % [5, 6]. Each group was assessed for statistically significant nodule growth, repeat FNA, thyroidectomy, and risk of malignancy using χ^2^ tests and logistic regression analyses. These analyses were corrected for age and sex.

Finally, acknowledging that growth was an important reason for repeat FNA and thyroidectomy, we sought to identify the determinants of benign nodule growth. To do so, the effects of patient (age, sex) and nodule characteristics (single versus multinodular, size, and solid versus cystic parenchyma) on growth were tested using linear and logistic regression analyses, correcting for follow-up time. Sensitivity analyses were performed for the continuous variables to identify the cut-off with the strongest effects. Statistical analysis was performed using SPSS version 22 (SPSS IBM, New York), and *P* values <0.05 were considered significant.

## Results

Over a 10 year period, there were 34 patients with follow-up periods <6 months, and 88 patients who had initial surgery as the nodule was large (mostly >5 cm). There were no thyroidectomies because of suspicious US features or family history of thyroid carcinoma. In total, 1,372 patients with 2,006 cytologically benign thyroid nodules met entry criteria and were analyzed. We excluded patients aged <20 years (n = 43 nodules), given the uniqueness of this population and potential selection bias [18]. Overall, 144 separate nodules were excluded because initial follow-up was less than 6 months. This resulted in a final population of 1,819 nodules, as shown in Table 1. As expected, the population was predominantly female (89.8 %) with a mean age of 52.5 years. Thyroid nodule size averaged 2.1 cm. These baseline characteristics were not associated with follow-up time (all *P* >0.05).

**Table 1:** Baseline patient and nodule characteristics

Patients, n	1,254
Women, %	89
Age, years	52.5 (13.1)
Thyroid nodules, n	1,819
Maximum diameter, cm	2.1 (1.0)
Volume, cm^3^	3.7 (7.2)
Nodule characteristics, %:	
<50 % Cystic	88.5
≥50 % Cystic	11.5
Years from initial benign aspirate to first follow-up:	
Median (interquartile range)	1.4 (1.0–2.5) years
Range	0.5–14.1 years
0.5–1 year, n	489
1–2 years, n	715
2–3 years, n	249
3–4 years, n	143
>4 years, n	223

Age, maximum diameter and volume are shown as mean (SD)

The proportion of nodules identified in each follow-up quintile is as follows: 0.5–1 year follow-up, n = 489 nodules; >1–2 years follow-up, n = 715 nodules; >2–3 years follow-up, n = 249 nodules; >3–4 years follow-up, n = 143 nodules; >4 years follow-up, n = 223 nodules. Comparisons of the risk of nodule growth, repeat FNA or thyroidectomy, identification of a thyroid cancer (i.e. false-negative cytology), and thyroid cancer-attributable mortality are shown in Table 2. With longer follow-up intervals, more nodules demonstrated growth, using both the 15 % and 50 % volumetric cut-offs. This resulted in a greater number of repeat aspirations, though there were no differences in cytology results, even when follow-up duration was lengthened. The number of thyroidectomies similarly increased, most notably when the follow-up interval was longer than 3 years. Compressive symptoms were the predominant indication for thyroidectomy. Only seven malignancies were identified among 1,819 nodules, with no significant difference in malignancy risk between the cohorts. There were no (0 %) thyroid cancer related deaths during a median follow-up of 7.7 (interquartile range, 5.5–10.5) years.

**Table 2:** Time interval until first follow-up of a benign thyroid nodule and the risk of growth, repeat FNAs, thyroidectomies, malignancies and mortality

Follow-up time, years	n	15 % Growth, % (n)	50 % Growth, % (n)	Repeat FNAs, % (n)	Outcomes of repeat FNAs	Thyroidectomies, % (n)	Indication for thyroidectomy	Malignancies, % (n)	Disease- related mortality, % (n)
0.5–1	489	30.3 (148)	8.6 (42)	5.1 (25)	21 Benign 1 AUS 3 Non-diagnostic^a^	0.8 (4)	3 US Large size/growth 1 Compressive symptoms	0.2 (1)	0 (0)
>1–2	715	34.8 (249)	15.1 (108)	5.6 (40)	29 Benign 4 AUS 1 Susp. foll. neopl. 1 Susp. PTC 5 Non-diagnostic^b^	0.8 (6)	4 Abnormal repeat FNA 2 US Large size/growth	0.3 (2)	0 (0)
>2–3	249	40.2 (100)	19.7 (49)	8.8 (22)	18 Benign 1 AUS 1 Susp. foll. neopl. 1 Malignant 1 Non-diagnostic^c^	1.2 (3)	3 Abnormal repeat FNA	0.8 (2)	0 (0)
>3–4	143	50.3 (72)	34.3 (49)	18.9 (27)	22 Benign 3 AUS 2 Susp. foll. neopl.	4.9 (7)	3 Abnormal repeat FNA 2 Compressive symptoms 1 US Large size/growth 1 Afirma GEC positive	0.7 (1)	0 (0)
>4 (range 4.0–14.1)	223	52.5 (117)	35.0 (78)	19.3 (43)	35 Benign 3 AUS 1 Susp. Hurthle cell neopl. 1 Malignant 3 Non-diagnostic^d^	4.0 (9)	5 Compressive symptoms 2 Abnormal repeat FNA 1 US Large size/growth 1 Afirma GEC positive	0.4 (1)	0 (0)
*P* value		<0.0001	<0.0001	<0.0001		0.0001		0.77	–

^a^All nodules were >75 % cystic and had therefore a negligible low risk of malignancy and were not rebiopsied

^b^Three nodules were >75 % cystic and had therefore a negligible low risk of malignancy and were not rebiopsied. One nodule did not change in size during follow-up, and was therefore not rebiopsied. One nodule was surgically removed (lobectomy) due to its large size (4.4 cm) and histological diagnosis confirmed a 3.3 cm follicular variant PTC (see Table 3 subject no. 3)

^c^Nodule did not change in size during follow-up, and was therefore not rebiopsied

^d^One nodule >75 % cystic and another 50–75 % cystic, which had therefore a negligible low risk of malignancy. The third nodule underwent total thyroidectomy as this patient had another nodule diagnosed with malignant cytology. Histopathology confirmed a 1.1 cm follicular variant PTC, while the nodule with the non-diagnostic biopsy was histologically confirmed to be benign

FNA, Fine needle aspiration; AUS, Atypical cells of undetermined significance; PTC, Papillary thyroid carcinoma; GEC, Gene expression classifier. All malignancies were determined by histopathology and the malignancy percentage indicates the rate of malignancies for the respective follow-up time group

Table 3 shows the characteristics of the seven malignancies. All were follicular variants of papillary thyroid carcinoma (fvPTC). One patient (subject no. 1) underwent a thyroidectomy for a symptomatic nodule, and a fvPTC with focal capsular invasion and one scapular bone metastasis was diagnosed. The metastasis showed an excellent response to radioactive iodine treatment, and the patient is currently disease free. All other malignancies demonstrated very low risk characteristics. Specifically, all were encapsulated or partially-encapsulated/well-circumscribed, with only one nodule demonstrating extensive capsular invasion. None demonstrated evidence of lymphovascular invasion or extrathyroidal extension, and no lymph node or distant metastases were identified. All patients are presently alive and considered disease free.

**Table 3:** Description of the seven patients with false benign malignancies

Subject no.	Time until first follow-up, years	Nodule size at initial aspiration, cm	Nodule size at first follow-up, cm	Thyroidectomy indication	Histopathology	Encapsulated	Lymphovascular invasion/Extrathyroidal extension	Lymph node/Distant metastases	Clinical status
1	1.0	5.2	6.1	Compressive symptoms	PTC follicular variant 5.0 cm	Encapsulated ^a^	1 Focus suspicious for LVI	1 Scapular metastasis^c^	Disease free with no recurrence
2	1.3	4.0	4.5	Abnormal repeat cytology	PTC follicular variant 4.3 cm	Encapsulated	No	No	Disease free with no recurrence
3	1.4	2.9	4.4	Compressive symptoms	PTC follicular variant 3.3 cm	Encapsulated	No	No	Disease free with no recurrence
4	2.3	2.0	2.9	Abnormal repeat cytology	PTC follicular variant 3.8 cm	Encapsulated ^b^	No	No	Disease free with no recurrence
5	2.7	1.2	1.6	Abnormal repeat cytology	PTC follicular variant 1.1 cm	Encapsulated	No	No	Disease free with no recurrence
6	3.7	2.2	2.7	Abnormal repeat cytology	PTC follicular variant 2.5 cm	Encapsulated	No	No	Disease free with no recurrence
7	4.4	4.5	6.1	Abnormal repeat cytology	PTC follicular variant 5.5 cm	Partially-encapsulated/ well-circumscribed	No	No	Disease free with no recurrence

^a^With focal capsular invasion

^b^With extensive capsular invasion

^c^Metastasis showed excellent reaction to radioactive iodine treatment

PTC, Papillary thyroid carcinoma; LVI, Lymphovascular invasion

**Fig. 1:**
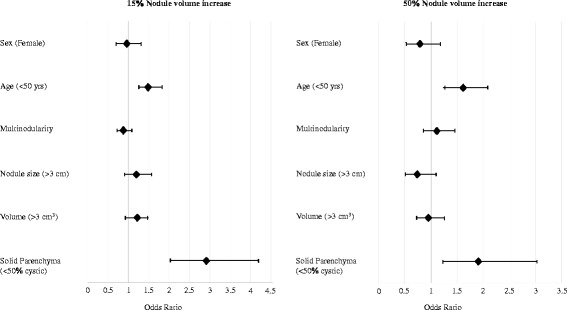
Determinants of benign nodule growth. Growth was calculated as the increase in volume between the baseline and first follow-up ultrasounds. All analyses were corrected for follow-up time, as well as for age and cystic content, as these factors were associated with growth in univariate analyses

**Fig. 2:**
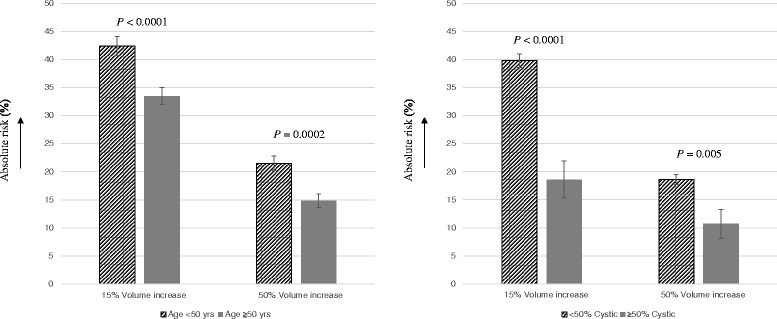
Effects of age <50 years and <50 % cystic content on the absolute risk of nodule growth. Growth was calculated as the increase in volume between the baseline and first follow-up ultrasounds. Age analyses were corrected for follow-up time, cystic content and sex, and cystic content analyses were corrected for follow-up time, age and sex

To further guide optimal follow-up strategies after a benign aspiration, we sought to identify determinants of benign nodule growth (Fig. 1). Sensitivity analyses were performed to identify the cut-off with the strongest effects. There were no effects of sex, multinodularity, or nodule size on the risk of growth when using the 15 % volume increase cut-off, nor when using the 50 % cut-off. However, an age <50 years was associated with a higher risk of nodule growth, both when using the 15 % cut-off (odd ratio (OR), 1.48; confidence interval (CI), 1.22–1.81; *P* <0.0001) as well as the 50 % cut-off (OR, 1.61; CI, 1.26–2.07; *P* = 0.0002). A solid parenchyma (<50 % cystic content) was also associated with a higher risk of growth, again both when using the 15 % (OR, 2.91; CI, 2.02–4.18; *P* <0.0001) and 50 % (OR, 1.91; CI, 1.21–3.02; *P* = 0.005) cut-offs. In order to provide more insight, Fig. 2 depicts the absolute risk of growth attributable to these factors.

## Discussion

This is the first study to investigate the optimal time interval for repeat evaluation of a benign thyroid nodule. While a longer time interval is associated with greater nodule growth, no detriments in health outcomes were identified. Nodule growth led to a higher risk of repeat FNA as well as thyroidectomy, but not an increased risk of malignancy. As an increased rate of thyroidectomy was only noted when follow-up exceeded 3 years, it appears that symptomatic concerns related to nodule growth were not clinically concerning with shorter follow-up intervals. Importantly, follow-up intervals exceeding even 4 years were not associated with a higher risk of detecting harmful malignancies or adverse consequences. The risk of false-negative cytology was similar among all cohorts, and malignancy-related mortality was 0 % in the entire study population. Together, these data show that most thyroid nodules with initial benign cytology can be safely recommended for repeat evaluation at a 3 year interval without increased risk or likelihood of harm.

Little data are available on the optimal time interval for repeat evaluation of a cytologically benign nodule. Negro et al. [19] described 249 patients with benign nodular disease who were followed for 5 years and documented the timing of relevant events, including the risk of repeat FNA, thyroidectomy or newly diagnosed thyroid malignancy. Unfortunately, due to the limited number of patients, this study was underpowered to stratify for follow-up time and did not investigate the outcomes at various time intervals. Separately, we have previously studied the disease-specific mortality attributable to thyroid malignancies in a pilot population of patients with cytologically benign nodules [7]. There were no malignancy-related deaths, while thyroidectomies were performed and malignancies identified at an average of 4.5 years. However, the design of that study was critically different from the present one as it did not investigate the outcomes of various follow-up intervals. It also did not investigate the risk of nodule growth, which is the driving factor for most relevant interventions, including repeat FNAs and thyroidectomies, as confirmed by the results of the present study.

Given this important role for growth, we also sought to find determinants of benign nodule growth in hopes that this may further guide the clinician in personalizing any follow-up interval using simple patient and US characteristics. Age under 50 years and a solid parenchyma were both associated with a substantially higher risk of growth. While our results support a broad recommendation for a 3 year follow-up interval, these sub-analyses suggest that a longer interval may be considered for older patients with more cystic nodules, or conversely slightly shorter intervals for young patients with solid nodules. Individual assessment incorporating all available data, including patient preferences, remains paramount. In this context it is of interest to note that the growth rates of the nodules which turned out to be histologically malignant did not differ from the growth rates of the nodules which were either cytologically (at repeat FNA) or histologically benign, with mean increases of 119.5 % (45.8) (mean (SE)) for the malignant and 108.5 % (10.3) for the benign nodules (*P* = 0.82). However, a borderline significant difference in their largest diameter at first visit was detected (3.1 (0.4) (mean (SE)) vs. 2.5 (0.9) cm, *P* = 0.08). Importantly, these data need to be interpreted with caution as only seven malignancies were detected in our cohort.

Recently, some studies have suggested that the presence of certain sonographic characteristics (such as hypoechogenicity, irregular margins or microcalcifications) should be used to determine the follow-up strategy of cytologically benign nodules [20, 21]. However, these studies are retrospective and based on a limited number of nodules with suspicious US characteristics (n = 93 and n = 102) [20, 21], while others were not able to replicate these findings [22]. Furthermore, various studies have shown that interobserver agreement for most of these characteristics is typically limited [23–26]. Thus, while helpful, it may therefore be difficult to use these specific US characteristics exclusively as a guide to optimal benign nodule follow-up. The exact role of these specific US characteristics in nodule care should therefore be determined in future studies.

Strengths of this study include its large sample size and the variation in time to first follow-up, which provides a robust comparison. We provide detailed data on sonographic growth, repeat FNAs, thyroidectomies, histopathological results and mortality, and additionally performed sub-analyses on determinants of benign nodule growth. However, we similarly acknowledge limitations to our study, including that it was performed in a single center. Despite this, our clinic captures more than 95 % of patients undergoing thyroid evaluation in our healthcare system, and all have been registered in our database, which improves the generalizability of our findings. While prospective, this study also does not pursue a randomized intervention. The main concern, therefore, would be that our follow-up intervals were not randomly determined but rather influenced by nodule-specific characteristics. However, this is unlikely given that all patients with benign thyroid nodules were provided similar recommendations for routine 1-year follow-up. Furthermore, long interval follow-ups were mostly attributable to patient compliance and organizational delays – factors unlikely to significantly bias our findings. Importantly, follow-up time intervals were not associated with nodule size, cystic content, age, or sex. It is therefore unlikely that the variation in follow-up intervals was related to nodule-specific factors. Furthermore, we cannot exclude that part of the patients with faster growth presented earlier. However, as previously discussed, growth did not seem to be a reliable indicator of malignancy risk. Finally, we intentionally only included results from the first follow-up visit. This is because management decisions made at subsequent follow-up visits are likely influenced by previous visits, while our goal was to investigate the clinical outcomes as a function of various time intervals through the first follow-up visit. While this was therefore the correct design for our main study outcomes, no recommendations on timing of further follow-up visits could be made.

## Conclusions

In summary, current guidelines recommend repeat evaluation of a benign thyroid nodule after 1–2 years, which is only based on expert opinion. This study suggests that this recommendation can be safely extended to 3 years without increased malignancy and mortality risk, or likelihood of harm. Nodule growth can be expected, and therefore young patients, those experiencing possible structural symptoms, or those with larger nodules at baseline may be candidates for modified recommendations. Conversely, even longer follow-up intervals should be considered for older patients with predominantly cystic nodules. While replication of these data in large prospective multicenter studies is needed, this extension in follow-up interval would reduce unnecessary visits and medical interventions for millions of affected patients worldwide, leading to healthcare savings.

### Availability of data and materials

These data are not publicly available as these concern patient-related data.

## References

[1] Sosa JA, Hanna JW, Robinson KA, Lanman RB (2013). Increases in thyroid nodule fine-needle aspirations, operations, and diagnoses of thyroid cancer in the United States. Surgery.

[2] Davies L, Welch HG (2014). Current thyroid cancer trends in the United States. JAMA Otolaryngol Head Neck Surg.

[3] Leenhardt L, Bernier MO, Boin-Pineau MH, Conte Devolx B, Maréchaud R, Niccoli-Sire P (2004). Advances in diagnostic practices affect thyroid cancer incidence in France. Eur J Endocrinol.

[4] Wang CC, Friedman L, Kennedy GC, Wang H, Kebebew E, Steward DL (2011). A large multicenter correlation study of thyroid nodule cytopathology and histopathology. Thyroid.

[5] Cooper DS, Doherty GM, Haugen BR, Kloos RT, Lee SL, American Thyroid Association Guidelines Taskforce on Thyroid Nodules and Differentiated Thyroid Cancer (2009). Revised American Thyroid Association management guidelines for patients with thyroid nodules and differentiated thyroid cancer. Thyroid.

[6] Gharib H, Papini E, Paschke R, Duick DS, Valcavi R, Hegedüs L (2010). American Association of Clinical Endocrinologists, Associazione Medici Endocrinologi, and European Thyroid Association medical guidelines for clinical practice for the diagnosis and management of thyroid nodules. Endocr Pract.

[7] Nou E, Kwong N, Alexander LK, Cibas ES, Marqusee E, Alexander EK (2014). Determination of the optimal time interval for repeat evaluation after a benign thyroid nodule aspiration. J Clin Endocrinol Metab.

[8] Alexander EK, Hurwitz S, Heering JP, Benson CB, Frates MC, Doubilet PM (2003). Natural history of benign solid and cystic thyroid nodules. Ann Intern Med.

[9] Durante C, Costante G, Lucisano G, Bruno R, Meringolo D, Paciaroni A (2015). The natural history of benign thyroid nodules. JAMA.

[10] Danese D, Sciacchitano S, Farsetti A, Andreoli M, Pontecorvi A (1998). Diagnostic accuracy of conventional versus sonography-guided fine-needle aspiration biopsy of thyroid nodules. Thyroid.

[11] Carmeci C, Jeffrey RB, McDougall IR, Nowels KW, Weigel RJ (1998). Ultrasound-guided fine-needle aspiration biopsy of thyroid masses. Thyroid.

[12] Morris LG, Sikora AG, Tosteson TD, Davies L (2013). The increasing incidence of thyroid cancer: the influence of access to care. Thyroid.

[13] Brito JP, Morris JC, Montori VM (2013). Thyroid cancer: zealous imaging has increased detection and treatment of low risk tumours. BMJ.

[14] Sosa JA, Hanna J, Lanman RB, Robinson KA, Ladenson PW. Increases in thyroid nodule fine needle aspirations, surgeries and diagnoses of thyroid cancer in the United States. American Association of Endocrine Surgeons 34^th^ Annual Meeting, Apr 14–16, 2013. Chicago, Ill. (oral abstract).

[15] Cibas ES (2009). Ali SZ; NCI Thyroid FNA State of the Science Conference. The Bethesda System for Reporting Thyroid Cytopathology. Am J Clin Pathol.

[16] Ali SZ, Cibas ES (2009). The Bethesda System for Reporting Thyroid Cytopathology.

[17] Brunn J, Block U, Ruf G, Bos I, Kunze WP (1981). Scriba PC. Dtsch Med Wochenschr.

[18] Gupta A, Ly S, Castroneves LA, Frates MC, Benson CB, Feldman HA (2013). A standardized assessment of thyroid nodules in children confirms higher cancer prevalence than in adults. J Clin Endocrinol Metab.

[19] Negro R (2014). What happens in a 5-year follow-up of benign thyroid nodules. J Thyroid Res.

[20] Kwak JY, Koo H, Youk JH, Kim MJ, Moon HJ, Son EJ (2010). Value of US correlation of a thyroid nodule with initially benign cytologic results. Radiology.

[21] Rosario PW, Purisch S (2010). Ultrasonographic characteristics as a criterion for repeat cytology in benign thyroid nodules. Arq Bras Endocrinol Metabol.

[22] Illouz F, Rodien P, Saint-André JP, Triau S, Laboureau-Soares S, Dubois S (2007). Usefulness of repeated fine-needle cytology in the follow-up of non-operated thyroid nodules. Eur J Endocrinol.

[23] Park SH, Kim SJ, Kim EK, Kim MJ, Son EJ, Kwak JY (2009). Interobserver agreement in assessing the sonographic and elastographic features of malignant thyroid nodules. AJR Am J Roentgenol.

[24] Park SJ, Park SH, Choi YJ, Kim DW, Son EJ, Lee HS (2012). Interobserver variability and diagnostic performance in US assessment of thyroid nodule according to size. Ultraschall Med.

[25] Park CS, Kim SH, Jung SL, Kang BJ, Kim JY, Choi JJ (2010). Observer variability in the sonographic evaluation of thyroid nodules. J Clin Ultrasound.

[26] Choi SH, Kim EK, Kwak JY, Kim MJ, Son EJ (2010). Interobserver and intraobserver variations in ultrasound assessment of thyroid nodules. Thyroid.

